# Occurrence of Extended-Spectrum and AmpC-Type β-Lactamase Genes in *Escherichia coli* Isolated from Water Environments in Northern Thailand

**DOI:** 10.1264/jsme2.ME17050

**Published:** 2017-09-27

**Authors:** Kanit Assawatheptawee, Uttapoln Tansawai, Anong Kiddee, Phetrada Thongngen, Phiraphat Punyadi, Tayawee Romgaew, Phattaraporn Kongthai, Tawatchai Sumpradit, Pannika R. Niumsup

**Affiliations:** 1 Department of Microbiology and Parasitology, Faculty of Medical Science, Naresuan University Phitsanulok, 65000 Thailand; 2 Center of Excellence in Medical Biotechnology, Faculty of Medical Science, Naresuan University Phitsanulok, 65000 Thailand

**Keywords:** *Escherichia coli*, *bla*_CTX-M_, *bla*_CMY-2_, water, ST131

## Abstract

Sixty-eight cefotaxime-resistant *Escherichia coli* isolates were recovered from different water environments in Northern Thailand. Isolates were mostly resistant to ceftazidime and aztreonam (>90%). The most common extended-spectrum β-lactamase-encoding gene was *bla*_CTX-M-group 1_ (75%) followed by *bla*_CTX-M-group 9_ (13.2%). The co-existence of *bla*_CTX-M_ and AmpC-type β-lactamase genes was detected in 4 isolates (5.9%). Two *E. coli* isolates carrying *bla*_CTX-M_ from canal and river water samples belonged to the phylogenetic group B2-ST131, which is known to be pathogenic. This is the first study on *bla*_CTX-M_ and *bla*_CMY-2_-carrying *E. coli* and the emergence of ST131 from water environments in Thailand.

*Escherichia coli* is a normal inhabitant of the intestinal tract of humans and various animals. Although some strains appear to be harmless, several pathogenic strains are regarded as important causes of community-associated urinary tract, gastrointestinal, and systemic infections in humans (9). The presence of *E. coli* strains in water environments suggests fecal contamination and poses a public health risk. The dissemination of antibiotic-resistant *E. coli* in different water environments is a serious public health issue worldwide, particularly in the Indochina region, including Thailand, in which sanitary services may be inadequate (15). Individuals may be infected by resistant bacteria from water environments, resulting in serious infections. Similar genotypes between antibiotic-resistant *E. coli* from water environments and human clinical isolates suggest the spread of these organisms to humans (5).

Extended-spectrum β-lactamases (ESBL), which mediate resistance to third generation cephalosporins and monobactams, are widely disseminated among Gram-negative bacteria from hospital settings and communities. Several types of ESBL have been reported, such as TEM and SHV derivatives, which evolved from a mutation in parental β-lactamase TEM-1 and -2 and SHV-1, respectively. Another type of ESBL, CTX-M β-lactamase, has become the most common ESBL found in *Enterobacteriaceae*. ESBLs have extended their ability to hydrolyze cefotaxime, ceftazidime, ceftriaxone, and aztreonam and are inhibited by β-lactamase inhibitors (clavulanic acid, tazobactam, and sulbactam) (13). Resistance to third generation cephalosporins may be caused by the production of AmpC β-lactamase, with CMY-2 being the most prevalent (13). Cephalosporin resistance mediated by the AmpC enzyme includes mutations within regulatory regions, resulting in the permanently strong expression of AmpC and acquisition of a transferable plasmid-mediated AmpC β-lactamase (13).

To date, data on antibiotic-resistant *E. coli*, particularly those producing ESBL and AmpC, have frequently been obtained from various water environments, such as rivers, lakes, wastewater, tap water, and drinking water, in different regions across continents, but are more abundant from developing countries (1, 12). ESBL- and AmpC-carrying *E. coli* isolates have been widely disseminated in Thailand for many years (7, 14). Several ESBL- and AmpC-encoding genes have been identified; however, most studies have primarily focused on clinical isolates. Information on antibiotic-resistant *E. coli* from non-hospital sources, particularly water environments, is limited. A previous study in Thailand revealed the presence of ESBL-producing *Enterobacteriaceae* in different water environments; however, the resistant determinants and the genotypes of resistant isolates have not been reported (2). The carriage rate of these organisms among the healthy Thai population was reported to be high (2). Furthermore, approximately 22% of Swedish travelers returning from Thailand developed diarrhea caused by ESBL-producing *E. coli* (17). Hence, ESBL-producing *E. coli* may be present in various environments in Thailand. This study was conducted in order to clarify whether water environments in Thailand serve as reservoirs for antibiotic-resistant *E. coli*, particularly those producing ESBL and AmpC.

This study was conducted in the Phitsanulok and Nakhon Sawan provinces, two fast-growing cities in Northern Thailand. These areas have a relatively high population density and are regarded as active regions for livestock and aquaculture farming. Municipal water, mainly obtained from water resources within these cities, is used as a household water supply. Between August 2013 and January 2014, water samples were collected from 33 different water resources in the study area (river, 4; pond, 6; canal, 6; tap water, 8 and groundwater, 9). Sampling sites were shown in Fig. 1. Water samples were collected from 30 cm below the water surface using sterile bottles (500 mL bottle^−1^, 3 bottles sampling site^−1^). Groundwater was obtained by pumping water from drilling wells, while tap water was collected directly from a tap; approximately the first 500 mL was discarded in each case. All samples were kept on ice, transported to the laboratory, and processed within 24 h of collection. Ten milliliters of water samples were enriched in 90 mL EE broth (Becton, Dickinson and Company, NJ, USA) at 37°C for 24 h. Enrichment cultures were then 10-fold serially diluted, spread on MacConkey agar (Oxoid, Hampshire, UK) supplemented with 2 mg L^−1^ cefotaxime (Sigma-Aldrich, MO, USA), and incubated at 37°C for 24–48 h. Approximately 5–10 colonies with a typical *E. coli* morphology per sample were picked and sub-cultured on Tryptic Soy agar (Oxoid). In order to avoid working with clones of the same *E. coli* strain, an enterobacterial repetitive intergenic consensus polymerase chain reaction (ERIC-PCR) was performed as described previously (18). Isolates were considered to belong to the same clone if they shared the same ERIC-PCR pattern. Isolates representing distinct DNA patterns were selected for subsequent studies. Species identification was performed using standard biochemical tests (Gram staining, growth on EMB, oxidase test) and 16S rRNA gene sequencing (8). Among 33 water resources, cefotaxime-resistant *E. coli* isolates were found in samples from different rivers (*n*=4), ponds (*n*=5), and canals (*n*=6) (Fig. 1, Table 1). Two tap water samples yielded cefotaxime-resistant isolates, while no isolates were detected from groundwater samples. Sixty-eight isolates were recovered and antimicrobial susceptibility testing was performed using the disk diffusion method, as recommended by the Clinical and Laboratory Standards Institute (4). An isolate was defined as being multidrug resistance (MDR) if it was resistant to three or more classes of antimicrobial agents. Most isolates were resistant to ampicillin, cefotaxime, ceftazidime, aztreonam, and streptomycin (>90%) (Fig. 2, Table S1). High rates of resistance to kanamycin, tetracycline, chloramphenicol, ciprofloxacin, and trimethoprim-sulfamethoxazole (>60%) were also noted. It is a matter of concern that all 68 *E. coli* isolates showed the MDR phenotype and 58.8% of isolates exhibited resistance to up to ten or more of the antimicrobial agents tested. The Minimum Inhibitory Concentrations (MICs) of the selected antibiotics were assessed using the broth microdilution method according to CLSI guidelines (4). High-level resistance to cefotaxime (MIC_90_>128 mg L^−1^), ceftazidime (MIC_90_=128 mg L^−1^), and ciprofloxacin (MIC_90_=32 mg L^−1^) was noted. In contrast, low MIC_90_ values were observed for imipenem (4 mg L^−1^) and meropenem (2 mg L^−1^).

The detection of genes coding for ESBL (*bla*_TEM_, *bla*_SHV_, and *bla*_CTX-M_ series) and AmpC (MOX, CIT, DHA, ACC, EBC, and FOX types) was performed by multiplex PCR using specific primers and conditions as previously described (6, 10, 19). Nucleotide sequences were analyzed with software available from the National Center for Biotechnology Information website (http://www.ncbi.nlm.nih.gov) (Table S1). In this study, as many as 88.2% (60/68) of cefotaxime-resistant *E. coli* isolates carried genes encoding for ESBL and AmpC (Table 1). Fifty-one (75%, 51/68) and 9 isolates (13.2%, 9/68) carried *bla*_CTX-M-group 1_ and *bla*_CTX-M-group 9_, respectively. The results of the sequence analysis revealed that the most common *bla*_CTX-M-group 1_ was *bla*_CTX-M-55_ (74.5%, 38/51) followed by *bla*_CTX-M-15_ (25.5%, 13/51). *bla*_CTX-M_ encoding for CTX-M-14 was found in all *bla*_CTX-M-group 9_-carrying isolates. These results were consistent with *bla*_CTX-M-14_, *bla*_CTX-M-15_, and *bla*_CTX-M-55_ being detected worldwide and commonly being found in Thai patients (7, 14). No *bla*_TEM_ or *bla*_SHV_-related ESBL genes were detected. However, the broad-spectrum β-lactamase gene, *bla*_TEM-1_, was found in 41 *bla*_CTX-M_-carrying isolates. Although *bla*_TEM-1_ is considered to be a non-ESBL, the hyperproduction of TEM-1 with a change in outer membrane proteins may result in reduced susceptibility to cefotaxime (20). Furthermore, 4 cefotaxime-resistant *E. coli* isolates (4/68, 5.9%) were positive for CIT-type AmpC genes. The results of the sequence analysis showed that all were *bla*_CMY-2_, which was found in combination with *bla*_CTX-M_. The emergence of carbapenem-resistant *E. coli* from water environments was observed (Fig. 2). The detection of carbapenemase genes (*bla*_IMP_, *bla*_VIM_, *bla*_NDM_, *bla*_KPC_, and *bla*_OXA_) by multiplex PCR was performed (11); however, the results obtained were negative. Carbapenem resistance may be due to the production of other carbapenemases, alterations in outer membrane proteins, and efflux pump overexpression (13).

The 68 cefotaxime-resistant *E. coli* isolates were classified into phylogenetic groups A, B1, B2, or D by multiplex PCR of the genes *chuA* and *yjaA* and the DNA fragment TspE4C2, as previously described (3). These results showed that most isolates belonged to the commensal groups B1 (55.9%, 38/68) and A (36.8%, 25/68). A small number of isolates belonged to the pathogenic groups B2 (2.9%, 2/68) and D (4.4%, 3/68) (Table S1). The recent global spread of the multi-resistant and highly virulent extraintestinal *E. coli* B2-sequence type (ST) 131 has been observed (9). We performed the MLST analysis by the amplification and sequencing of 7 housekeeping genes (*adk*, *fumC*, *gyrB*, *icd*, *mdh*, *purA*, and *recA*) according to the protocols on the *E. coli* MLST website (http://mlst.warwick.ac.uk/mlst/dbs/Ecoli). The results obtained showed that 2 *E. coli* isolates belonging to group B2 were identified as *E. coli* ST131. Both isolates showed resistance to the third generation cephalosporins, aztreonam and ciprofloxacin, and carried *bla*_CTX-M_. The amplification and sequence analysis of full-length *bla*_CTX-M_ revealed that one isolate carried *bla*_CTX-M-14_, while the other carried *bla*_CTX-M-27._

The 2 *E. coli* ST131 isolates were typed by pulsed field gel electrophoresis (PFGE). The preparation of chromosomal DNA in agarose plugs and digestion with *Xba*I (Fermentas, NY, USA) were performed as described previously (21). DNA was electrophoresed through 1% Pulsed Field Certified agarose in 0.5×TBE (Tris–borate–EDTA) buffer (CHEF Mapper XA System, Bio-Rad Laboratories, CA, USA). Banding patterns were interpreted by visual inspection. We observed that both ST131 isolates were closely related (<3 band differences) according to the criteria defined by Tenover *et al.* (16) (Fig. 3). These results suggested that the 2 ST131 isolates originated from the same clone even though they were obtained from distantly related water resources (approximately 20 km) (Fig. 1). One ST131 isolate (*bla*_CTX-M-14_-positive) was recovered from a canal outside the city, near to which many agricultural and farming activities were being conducted, while the other (*bla*_CTX-M-27_-positive) was obtained from a river located in the residential area within the city. To date, the presence of *E. coli* ST131 in humans, animals, and food products has been documented; however, limited information is currently available on the prevalence of ST131 in water environments (9). Previous studies in European countries identified ST131 in water environments (5, 22). However, studies in Asian countries, such as India and Bangladesh, in which ESBL-producing *E. coli* isolates from water environments were studied, did not identify ST131 (1, 12). Our results suggested that water environments in Thailand contained virulent and MDR *E. coli* that have the potential to cause serious infections.

Our results of MDR *E. coli* carrying ESBL and AmpC from different water environments were similar to those reported from other regions, even in countries that have good water supply and sanitation services, such as Switzerland (22). These results were not unexpected because of the inappropriate use of antimicrobial agents for chemotherapy, livestock farming, and aquaculture (15). Moreover, antibiotics are easily obtained without a prescription in Thailand. Antimicrobial-resistant bacteria from human activities may easily be released into water environments and subsequently lead to the dissemination of these organisms within the community.

In conclusion, this is the first study on *bla*_CTX-M_- and *bla*_CMY-2_-carrying *E. coli* from different water environments in Thailand. The matter of most concern is the emergence of *bla*_CTX-M_-carrying *E. coli* ST131 due to its ability to cause severe infections. Our results have important implications for the health of individuals living in the community. Furthermore, these results highlight the need for the more extensive surveillance of water environments in Thailand.

The nucleotide sequences of *bla*_CTX-M_ and *bla*_CMY-2_ reported in this study were deposited in the GenBank database under the accession numbers MF374734–MF374735, MF385034–MF385037, MF406113–MF406132, and MF422526.

## Supplementary Material



## Figures and Tables

**Fig. 1:**
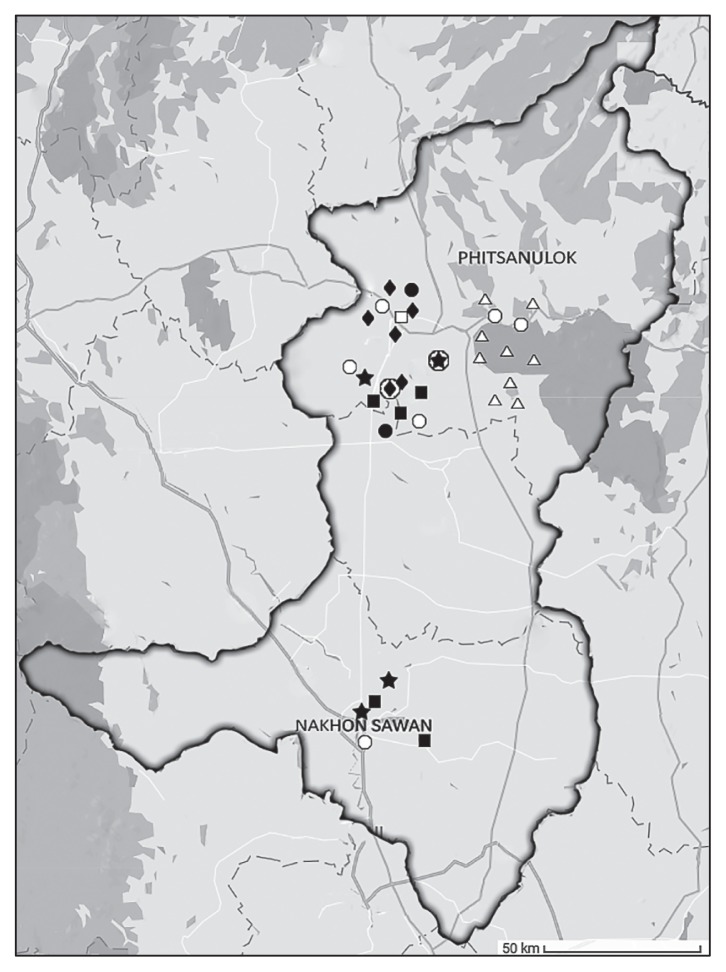
Location of water sampling. Black and white symbols represented sampling sites that were positive and negative for cefotaxime-resistant *E. coli*, respectively. Star, river; square, pond; diamond, canal; circle, tap water; triangle, groundwater. Circled diamond and star represent water environments that were positive for *bla*_CTX-M-14_- and *bla*_CTX-M-27_-carrying *E. coli* ST131, respectively.

**Fig. 2:**
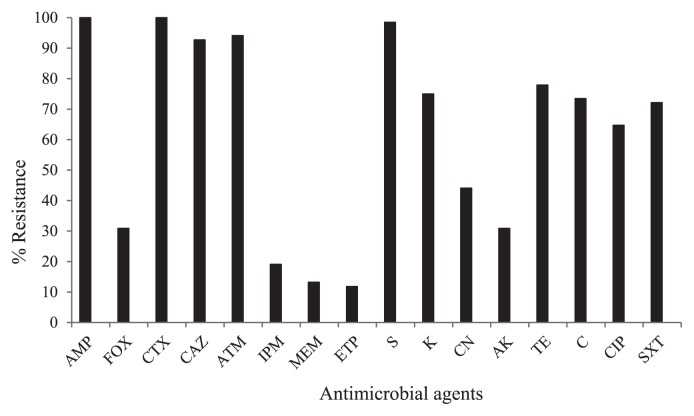
Occurrence ratio (%) of antimicrobial-resistant *E. coli* isolates. Antimicrobial resistance was assessed using the disk diffusion method according to the CLSI guidelines (4). AMP, ampicillin; FOX, cefoxitin; CTX, cefotaxime; CAZ, ceftazidime; ATM, aztreonam; IPM, imipenem; MEM, meropenem; ETP, ertapenem; S, streptomycin; K, kanamycin; CN, gentamicin; AK, amikacin; TE, tetracycline; C, chloramphenicol; CIP, ciprofloxacin, and SXT, trimethoprim-sulfamethoxazole.

**Fig. 3:**
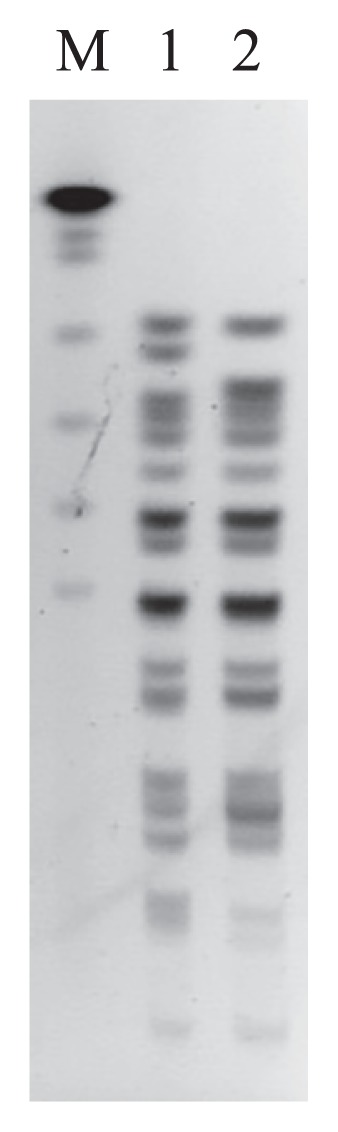
PFGE profiles of *bla*_CTX-M_-carrying *E. coli* ST131 isolated from different water environments in Phitsanulok province. M, *Saccharomyces cerevisiae* chromosomal DNA (Bio-Rad); lane 1, *bla*_CTX-M-14_-carrying *E. coli* ST131 isolated from a canal outside the city; lane 2, *bla*_CTX-M-27_-carrying *E. coli* ST131 isolated from a river within the city.

**Table 1:** Occurrence of cefotaxime-resistant *E. coli* from various water environments and the presence of β-lactamase genes.

Different water environments (No. of sampling sites where *E. coli* were isolates)	No. of *E. coli* isolates (*n*=68)	β-lactamase genes						

		none	*bla*_CTX-M- 1_	*bla*_CTX-M- 1+_ *bla*_TEM-1_	*bla*_CTX-M- 1+_ *bla*_CMY-2_	*bla*_CTX-M- 9_	*bla*_CTX-M- 9+_ *bla*_TEM-1_	*bla*_CTX-M- 9+_ *bla*_CMY-2_
River (*n*=4)	20	2	3	9	0	3	1	2
Pond (*n*=5)	16	2	4	9	1	0	0	0
Canal (*n*=6)	26	4	1	18	1	2	0	0
Tap water (*n*=2)	6	0	1	4	0	1	0	0
Total	68	8	9	40	2	6	1	2
