# Supplementary material for: Occurrence of Extended-Spectrum and AmpC-Type β-Lactamase Genes in Escherichia coli Isolated from Water Environments in Northern Thailand
Source: Microbes Environ. 2017 Sep 27;32(3):293–6. doi: 10.1264/jsme2.ME17050 (PMC5606701; doi:10.1264/jsme2.ME17050)
Supplement: Supplementary file 1 [file 32_293_s1.pdf]

Table S1. Isolation sources and characteristics of 68 *Escherichia coli* isolates recovered from water environments in Northern Thailand.

No.	Isolation source	City	Resistant profile	ß-lactamase genes	Phylogenetic group
1	pond	Phitsanulok	AmpCtxCazAtmSAkKCipTeSxt	*bla*$_{\text{CTX-M group 1}}$	B1
2	pond	Phitsanulok	AmpCtxCazAtmFoxSCnKCipTeSxt	*bla*$_{\text{CTX-M group 1}}$	D
3	pond	Phitsanulok	AmpCtxCazAtmImpSCCip	*bla*$_{\text{CTX-M group 1}}$	B1
4	pond	Phitsanulok	AmpCtxCazSKTeSxt	*bla*$_{\text{CTX-M group 1}}$	A
5	pond	Phitsanulok	AmpCtxCazAtmFoxImpSAkK	*bla*$_{\text{CTX-M group 1}}$+ *bla*$_{\text{CMY-2}}$	A
6	pond	Phitsanulok	AmpCtxCazAtmImpSKCCip	*bla*$_{\text{CTX-M group 1}}$	B1
7	pond	Phitsanulok	AmpCtxCazAtmSKCCip	*bla*$_{\text{CTX-M group 1}}$	B1
8	pond	Phitsanulok	AmpCtxCazAtmFoxEtpMemSCTeSxt	–	A
9	canal	Phitsanulok	AmpCtxCazAtmSCCip	*bla*$_{\text{CTX-M group 1}}$	B1
10	canal	Phitsanulok	AmpCtxCazAtmFoxSK	*bla*$_{\text{CTX-M group 1}}$+ *bla*$_{\text{CMY-2}}$	A
11	canal	Phitsanulok	AmpCtxCazAtmSCnAkKTe	*bla*$_{\text{CTX-M group 1}}$	B1
12	canal	Phitsanulok	AmpCtxCazAtmSCnAkKTe	*bla*$_{\text{CTX-M group 1}}$	B1
13	canal	Phitsanulok	AmpCtxCazAtmSCnAkKTe	*bla*$_{\text{CTX-M group 1}}$	B1
14	canal	Phitsanulok	AmpCtxCazAtmFoxImpEtpMemSCSxt	–	A
15	canal	Phitsanulok	AmpCtxAtmSKCip	*bla*$_{\text{CTX-M group 9}}$	B2
16	canal	Phitsanulok	AmpCtxCazAtmFoxEtpMemSCSxt	–	A
17	Tap water	Phitsanulok	AmpCtxCazAtmFoxSKTe	*bla*$_{\text{CTX-M group 9}}$	A
18	Tap water	Phitsanulok	AmpCtxCazAtmSKCTe	*bla*$_{\text{CTX-M group 1}}$	A
19	river	Phitsanulok	AmpCtxCazAtmSCTeSxt	*bla*$_{\text{CTX-M group 1}}$	A
20	river	Phitsanulok	AmpCtxCazAtmFoxSKTeSxt	*bla*$_{\text{CTX-M group 9}}$	A
21	river	Phitsanulok	AmpCtxCazAtmSCip	*bla*$_{\text{CTX-M group 9}}$	B2
22	river	Phitsanulok	AmpCtxCazAtmSCTe	*bla*$_{\text{CTX-M group 1}}$	B1
23	river	Phitsanulok	AmpCtxSKTe	*bla*$_{\text{CTX-M group 9}}$	A
24	river	Phitsanulok	AmpCtxCazAtmFoxIpmEtpMemSCnAkCCipTeSxt	–	A
25	river	Phitsanulok	AmpCtxCazAtmFoxIpmEtpMemSCSxt	–	A
26	canal	Phitsanulok	AmpCtxCazAtmSAkKTeSxt	*bla*$_{\text{CTX-M group 9}}$	A

27	canal	Phitsanulok	AmpCtxCazAtmFoxIpmEtpMemSCSxt	–	A
28	canal	Phitsanulok	AmpCtxCazAtmSCTeSxt	*bla*$_{CTX\text{-}M\ group\ 1}$	B1
29	pond	Nakhonsawan	AmpCtxCazAtmSCnKCCipTeSxt	*bla*$_{CTX\text{-}M\ group\ 1}$	B1
30	pond	Nakhonsawan	AmpCtxCazAtmSCnKCCipTeSxt	*bla*$_{CTX\text{-}M\ group\ 1}$	B1
31	pond	Nakhonsawan	AmpCtxAtmFoxIpmEtpMem	–	A
32	pond	Nakhonsawan	AmpCtxCazAtmSCnAkKCCipTeSxt	*bla*$_{CTX\text{-}M\ group\ 1}$	B1
33	pond	Nakhonsawan	AmpCtxCazAtmSCnKCCipTeSxt	*bla*$_{CTX\text{-}M\ group\ 1}$	B1
34	pond	Nakhonsawan	AmpCtxCazAtmSKCCipTeSxt	*bla*$_{CTX\text{-}M\ group\ 1}$	B1
35	pond	Nakhonsawan	AmpCtxAtmSCnKCCipTeSxt	*bla*$_{CTX\text{-}M\ group\ 1}$	B1
36	pond	Nakhonsawan	AmpCtxCazAtmFoxSCnAkKCCipTeSxt	*bla*$_{CTX\text{-}M\ group\ 1}$	B1
37	river	Nakhonsawan	AmpCtxCazAtmSKCCipTeSxt	*bla*$_{CTX\text{-}M\ group\ 1}$	B1
38	river	Nakhonsawan	AmpCtxCazAtmFoxSCnAkKCCipTeSxt	*bla*$_{CTX\text{-}M\ group\ 9}$+ *bla*$_{CMY\text{-}2}$	A
39	river	Nakhonsawan	AmpCtxCazAtmSKCCipTeSxt	*bla*$_{CTX\text{-}M\ group\ 1}$	B1
40	river	Nakhonsawan	AmpCtxCazFoxIpmSAkK	*bla*$_{CTX\text{-}M\ group\ 1}$	A
41	river	Nakhonsawan	AmpCtxCazAtmFoxSCnAkKCCipTeSxt	*bla*$_{CTX\text{-}M\ group\ 9}$+ *bla*$_{CMY\text{-}2}$	D
42	river	Nakhonsawan	AmpCtxCazAtmSCnAkKCCipTeSxt	*bla*$_{CTX\text{-}M\ group\ 1}$	A
43	river	Nakhonsawan	AmpCtxCazAtmSCnAkKCCipTeSxt	*bla*$_{CTX\text{-}M\ group\ 1}$	B1
44	river	Phitsanulok	AmpCtxCazAtmFoxSCnAkKCCipTeSxt	*bla*$_{CTX\text{-}M\ group\ 1}$	B1
45	river	Phitsanulok	AmpCtxCazAtmFoxSCnKCCipTeSxt	*bla*$_{CTX\text{-}M\ group\ 1}$	B1
46	river	Phitsanulok	AmpCtxCazAtmSCnAkKCCipTeSxt	*bla*$_{CTX\text{-}M\ group\ 1}$	B1
47	canal	Phitsanulok	AmpCtxCazAtmSCnKCCipTeSxt	*bla*$_{CTX\text{-}M\ group\ 1}$	B1
48	canal	Phitsanulok	AmpCtxCazAtmSKCCipTeSxt	*bla*$_{CTX\text{-}M\ group\ 1}$	B1
49	canal	Phitsanulok	AmpCtxCazAtmSCnAkKCCipTeSxt	*bla*$_{CTX\text{-}M\ group\ 1}$	B1
50	canal	Phitsanulok	AmpCtxCazAtmIpmSCnAkKCCipTeSxt	*bla*$_{CTX\text{-}M\ group\ 1}$	B1
51	canal	Phitsanulok	AmpCtxCazAtmSCnKCCipTeSxt	*bla*$_{CTX\text{-}M\ group\ 1}$	B1
52	canal	Phitsanulok	AmpCtxCazAtmFoxSCnAkKCCipTeSxt	*bla*$_{CTX\text{-}M\ group\ 1}$	B1
53	canal	Phitsanulok	AmpCtxCazAtmFoxEtpMemSCSxt	–	A
54	canal	Phitsanulok	AmpCtxCazAtmSCnKCCipTeSxt	*bla*$_{CTX\text{-}M\ group\ 1}$	B1
55	canal	Phitsanulok	AmpCtxCazAtmSCnKCCipTeSxt	*bla*$_{CTX\text{-}M\ group\ 1}$	B1

56	canal	Phitsanulok	AmpCtxCazAtmFoxIpmSCnKCCipTeSxt	*bla*$_{\text{CTX-M group 1}}$	B1
57	canal	Phitsanulok	AmpCtxCazAtmSCnAkKCCipTeSxt	*bla*$_{\text{CTX-M group 1}}$	B1
58	canal	Phitsanulok	AmpCtxCazAtmMemSKCTeSxt	*bla*$_{\text{CTX-M group 1}}$	A
59	canal	Phitsanulok	AmpCtxCazAtmIpmSCnKCCipTeSxt	*bla*$_{\text{CTX-M group 1}}$	A
60	canal	Phitsanulok	AmpCtxCazAtmIpmSCnAkKCCipTeSxt	*bla*$_{\text{CTX-M group 1}}$	A
61	canal	Phitsanulok	AmpCtxCazAtmSCCipTeSxt	*bla*$_{\text{CTX-M group 1}}$	B1
62	Tap water	Phitsanulok	AmpCtxCazAtmSKCCipTeSxt	*bla*$_{\text{CTX-M group 1}}$	A
63	Tap water	Phitsanulok	AmpCtxCazAtmSCCipTeSxt	*bla*$_{\text{CTX-M group 1}}$	B1
64	Tap water	Phitsanulok	AmpCtxCazAtmSCnKCCipTeSxt	*bla*$_{\text{CTX-M group 1}}$	B1
65	Tap water	Phitsanulok	AmpCtxCazAtmSKCCipTeSxt	*bla*$_{\text{CTX-M group 1}}$	B1
66	river	Nakhonsawan	AmpCtxCazAtmSCipTe	*bla*$_{\text{CTX-M group 1}}$	B1
67	river	Nakhonsawan	AmpCtxSKCCipTeSxt	*bla*$_{\text{CTX-M group 9}}$	D
68	river	Nakhonsawan	AmpCtxCazAtmSKTe	*bla*$_{\text{CTX-M group 1}}$	A

Abbreviations: AMP, ampicillin; CTX, cefotaxime; CAZ, ceftazidime; ATM, aztreonam; FOX, cefoxitin; IPM, imipenem; MEM, meropenem; ETP, ertapenem; S, streptomycin; CN, gentamicin; AK, amikacin; K, kanamycin; C, chloramphenicol; TE, tetracycline; CIP, ciprofloxacin and SXT, trimethoprim-sulfamethoxazole.
